# Generation of liver metastases in a mouse model using ultrasound-guided intravenous injection

**DOI:** 10.1016/j.xpro.2023.102163

**Published:** 2023-03-16

**Authors:** Amanda Labora, Hailey Lee, Charlotte Chan, Erin Tabornal, Thuc Le, Khalid Rashid, Evan Abt, Takanobu Yamao, Hanna Mandl, Amanda Creech, Alykhan Premji, Luyi Li, Jason Link, Nanping Wu, Caius Radu, Timothy Donahue

**Affiliations:** 1 Department of Surgery, University of California, Los Angeles, Los Angeles, CA 90095, USA; 2 Department of Molecular and Medical Pharmacology, Ahmanson Translational Imaging Division, University of California, Los Angeles, Los Angeles, CA 90095, USA

**Keywords:** Cancer, Cell Biology, Health Sciences, Model Organisms

## Abstract

Here, we present a protocol to generate a murine model of liver metastasis by directly injecting tumor cells into the portal vein under ultrasound guidance. We describe steps for animal and cell preparation and two techniques for injecting tumor cells. One technique is freehand, while the other technique is device-assisted using a 3D-printed prototype device. Finally, we describe tumor surveillance with bioluminescent imaging.

## Highlights

• Ultrasound-guided seeding of tumor cells in the mouse liver to generate liver metastases
• Injection of tumor cells into the portal vein with freehand or device-assisted technique
• Development of a prototype device to enhance injection stability
• Applicable to various tumor types and in immunocompetent or immunodeficient hosts

## Abstract (editor highlights)

Publisher’s note: Undertaking any experimental protocol requires adherence to local institutional guidelines for laboratory safety and ethics.

## Graphical abstract

**Figure:**
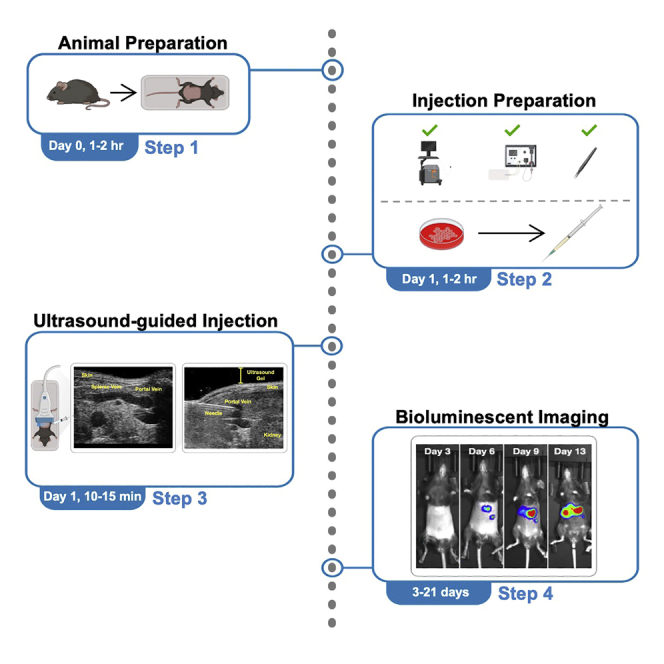

## Before you begin

This protocol describes steps for generating a liver metastatic model of melanoma with luciferase-expressing B16F10 (B16F10-luc) cells in C57BL/6 mice and employs bioluminescent imaging (BLI) for surveillance of tumor burden. This protocol was also successfully validated in another tumor model with the luciferase-expressing human pancreatic cancer cell line HPAC (HPAC-luc) in immunocompromised NCG mice. Additionally, this protocol describes how to perform the procedure using a needle-stabilization device. This procedure can be performed with or without the device (freehand), though the latter approach is more challenging. Approval from the appropriate animal research review board should be obtained prior to conducting any experiments involving live vertebrates.

### Institutional permissions

All reported animal studies were approved by the University of California, Los Angeles, Institutional Animal Care and Use Committee (IACUC), ARC-2012-089.

### Cell culture preparation

**Timing: 5–7 days**

1. Thaw the tumor cells of interest and culture cells in the appropriate medium (DMEM with 10% FBS for B16F10-luc and DMEM/F12 (1:1) with 10% FBS for HPAC-luc).
2. Maintain cells in culture at 37°C in 5% CO_2_ and allow the cells to acclimate to culture conditions following the thawing process.
3. Prepare for injection when the preferred confluency is achieved.

***Note:*** We recommend reaching approximately 80% confluency prior to injecting cells. It is not recommended to use cells that are too confluent as this can compromise cell viability.

## Key resources table

**Table:** 

REAGENT or RESOURCE	SOURCE	IDENTIFIER
**Chemicals, peptides, and recombinant proteins**		

D-Luciferin, potassium salt	BioVision	7903
Fluriso (isoflurane, USP)	Vet One	NDC13985-528-60
PBS 1×	Corning	21-031-CM
0.25% Trypsin-EDTA 1×	Gibco	25200-056
DMEM	Corning	10017CM
DMEM/F12 (1:1)	Gibco	11330-032
Fetal bovine serum (FBS)	Omega Scientific	FB-01

**Experimental models: Cell lines**		

Murine: B16F10-luciferase (B16F10-luc), P3-P10	ATCC	CRL-6475
Murine: HPAC-luciferase (HPAC-luc), P3-P10	ATCC	CRL-2119

**Experimental models: Organisms/strains**		

Mouse: C57BL/6, aged 6–8 weeks, female	The Jackson Laboratory	JAX:000664
Mouse: NCG, aged 6–8 weeks, female	Charles River	572-NCG

**Software and algorithms**		

Living Images v. 4.5	PerkinElmer	128110

**Other**		

Vevo® 2100 Imaging System	VisualSonics	https://www.biotech.cornell.edu/sites/default/files/2020-06/Vevo%202100%20Operator%20Manual.pdf
Vevo® Imaging Station	VisualSonics	https://www.biotech.cornell.edu/sites/default/files/2020-06/Vevo%202100%20Operator%20Manual.pdf
MicroScan™ Transducer	VisualSonics	https://www.biotech.cornell.edu/sites/default/files/2020-06/Vevo%202100%20Operator%20Manual.pdf
∗Set screw	McMaster	92311A077
∗Screw	McMaster	91251A211
∗Nuts	McMaster	90480A005
∗Hex key, 0.035″	McMaster	7122A37
∗Hex key, 3/32″	McMaster	7122A16
∗Wrench, 1/4″	McMaster	7242A31
∗Prusa MKS3+ Printer	Prusa3D	https://www.prusa3d.com/product/original-prusa-i3-mk3s-kit-3/
∗PETG filament	Prusa3D	https://www.prusa3d.com/product/prusament-petg-jet-black-1kg-refill/
∗00044-001	NA	NA
∗00044-002	NA	NA
IsoTec 5 Isoflurane Vaporizer	Datex Ohmeda	N/A
Ultrasound gel	Medline	MDS092005
U-100 insulin syringes (31G)	EasyTouch	08496-3156-11
Sterile alcohol prep pads	Fisherbrand	22-363-750
Toothed forceps	N/A	N/A
Electric shaver	N/A	N/A
Gel cream hair remover	Veet	3116876
Cotton-tipped applicators	Fisherbrand	REF: 22363157
KimWipes™ Delicate Task Wipes	Kimtech	Code: 34155
Micropore surgical tape	3M	1530-0
Microcentrifuge tube, 1.5 mL	Thermo Scientific	3448
Stainless steel laboratory spatula	N/A	N/A
Artificial tears lubricant ophthalmic ointment	Henry Schein	NDC 11695-6832-1
Thermo-peep heated pad	K&H Pet Products	https://khpet.com/collections/small-animal-products/products/thermo-peep-heated-pad
Absorbent blue pad	Denville	1158J48

∗3D-printed prototype device supplies.

## Materials and equipment

***Note:*** The Vevo® 2100 Imaging System, Vevo® Imaging Station and MicroScan™ Transducer were manufactured by Visual Sonics. The Vevo® Imaging System refers to the ultrasound machine; the MicroScan™ Transducer is an individually purchased compatible probe. The Vevo® Imaging Station includes the animal handling and physiological monitoring system, which functions as a temperature-controlled heated platform, the integrated rail base, which includes the height control and forward/backward control knobs, and the transducer mounting system, which functions to secure the ultrasound probe. Visual Sonics no longer sells this model. The Vevo® F2 LT and Vevo® F2 are two alternative models. The link in the key resources table is to a comprehensive user manual for the Vevo® 2100 Imaging System, which also includes the Vevo® Imaging Station and MicroScan™ Transducer.

***Note:*** MicroScan Transducer is a critical material for this protocol.

### 3D-printed prototype device

**Figure 1:**
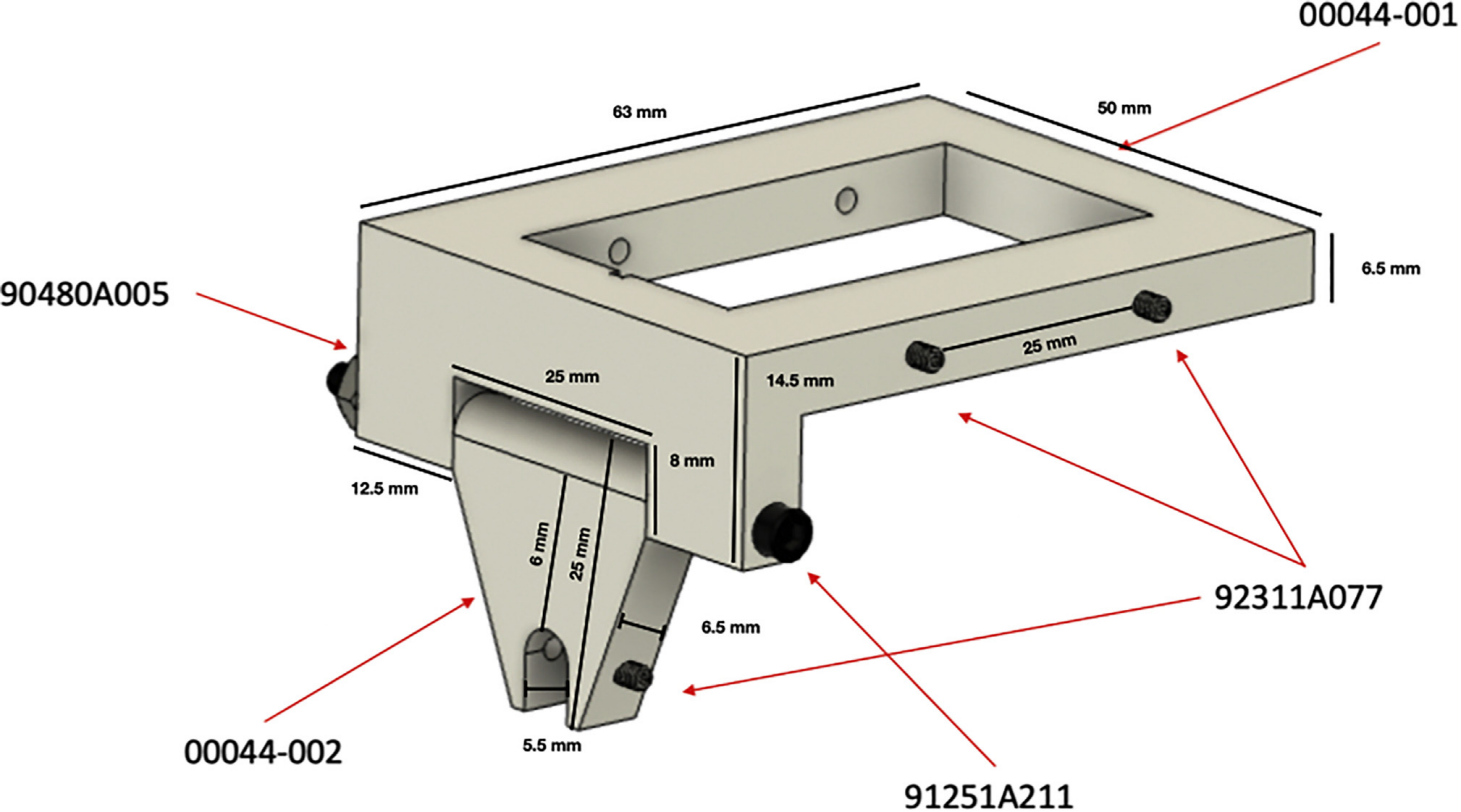
3D-printed prototype device schematic with dimensions and assembly instructions

To address the most challenging aspect of this protocol—maintaining the needle in plane with the ultrasound probe—we developed a 3D-printed prototype device that functions to maintain the syringe and needle in plane with the probe throughout the procedure. Refer to the key resources table for the list of the supplies used to print the device. Figure 1 illustrates device assembly.

## Step-by-step method details

### Animal preparation: Fur removal

**Timing: 5–10 min per mouse, 24 h prior to injection**

During this step, perform fur removal to prepare the animals for injection under ultrasound guidance.

***Note:*** Fur removal is performed at least 24 h prior to injection to minimize cumulative time under general anesthesia. If fur removal is performed too far in advance (more than 72 h), fur can grow back and interfere with image acquisition.

1. Prepare Depilatory Station.
  a. Turn on the heated platform and set to 37°C.
  b. Ensure that there is sufficient isoflurane and oxygen to carry out the procedure.
    ***Note:*** For the development of this protocol, 5% isoflurane in oxygen (5 liters per minute) was used for anesthesia induction and 2.5% isoflurane in oxygen (5 liters per minute) was used for anesthesia maintenance. Prior to starting the procedure, the anesthetic chamber was topped off with isoflurane.
  c. Review tubing connections between the anesthetic chamber and anesthetic delivery cone to ensure there are no leaks and that stopcocks are in the closed position (perpendicular to tubing).
    ***Note:*** Any previously approved anesthesia induction chamber and nose cone used for diagnostic imaging will work for this application. The purpose of this step is to minimize unnecessary exposure to isoflurane.
  d. Place an absorbent blue pad on the heated platform and assemble tools:
    i. Electric shaver.
    ii. Unscented depilatory cream.
    iii. PBS.
    iv. Metal spatula.
    v. Kimwipes.
    vi. Cotton-tipped applicators.
2. Induce Anesthesia.
  a. Transfer one mouse at a time to the anesthesia chamber and induce general anesthesia as per local animal review committee protocol.
  b. Apply ophthalmic ointment to both eyes for anesthetic events lasting longer than 5 min.
  c. Transfer anesthetized mouse to the preheated platform draped with an absorbent pad and continue anesthesia administration via nose cone.

***Optional:*** If an assistant is present to monitor mice under anesthesia, place the next mouse into the induction chamber.

3. Remove Fur.
  a. Using an electric shaver, remove fur from the anterior abdomen (from the costal margin to the mid abdomen).
  b. Dispense a dime-sized amount of unscented depilatory cream and spread evenly over the shaved area using a cotton-tipped applicator.
  c. Wait for approximately 2 min.
  d. Using a metal spatula, gently remove cream and fur until skin is completely fur-free.
    ***Note:*** Even fine hair patches produce sufficient artifact to prevent visualization of anatomic structures. Complete fur removal with depilatory cream is required for crisp, artifact-free imaging. Shaving removes most of the fur and makes the second step of fur removal with depilatory cream more efficient.
  e. Using a lightly moistened Kimwipe, remove excess depilatory cream.
  f. Pat the mouse skin dry with a dry Kimwipe.
  g. Transfer the mouse back to the cage on a heated platform and monitor for recovery from anesthesia.
  h. Repeat above steps until all mice have undergone hair removal and have recovered from anesthesia.

### Ultrasound imaging and injection station preparation

**Timing: 30 min**

During this step, prepare the imaging and injection station and check the experimental equipment prior to use.

4. Review tubing connections between the anesthetic chamber and anesthetic delivery cone.
5. Ensure that there is sufficient isoflurane and oxygen in the anesthesia induction chamber to carry out the procedure.

***Note:*** For the development of this protocol, 5% isoflurane in oxygen was used for anesthesia induction and 2.5% isoflurane in oxygen was used for anesthesia maintenance.

6. Turn on the physiological controller from the Vevo® Imaging Station and set the platform temperature to 37°C.

**CRITICAL:** The mice have previously undergone hair removal and are particularly susceptible to hypothermia. If the platform is not well heated, mice can die from hypothermia.

**Figure 2:**
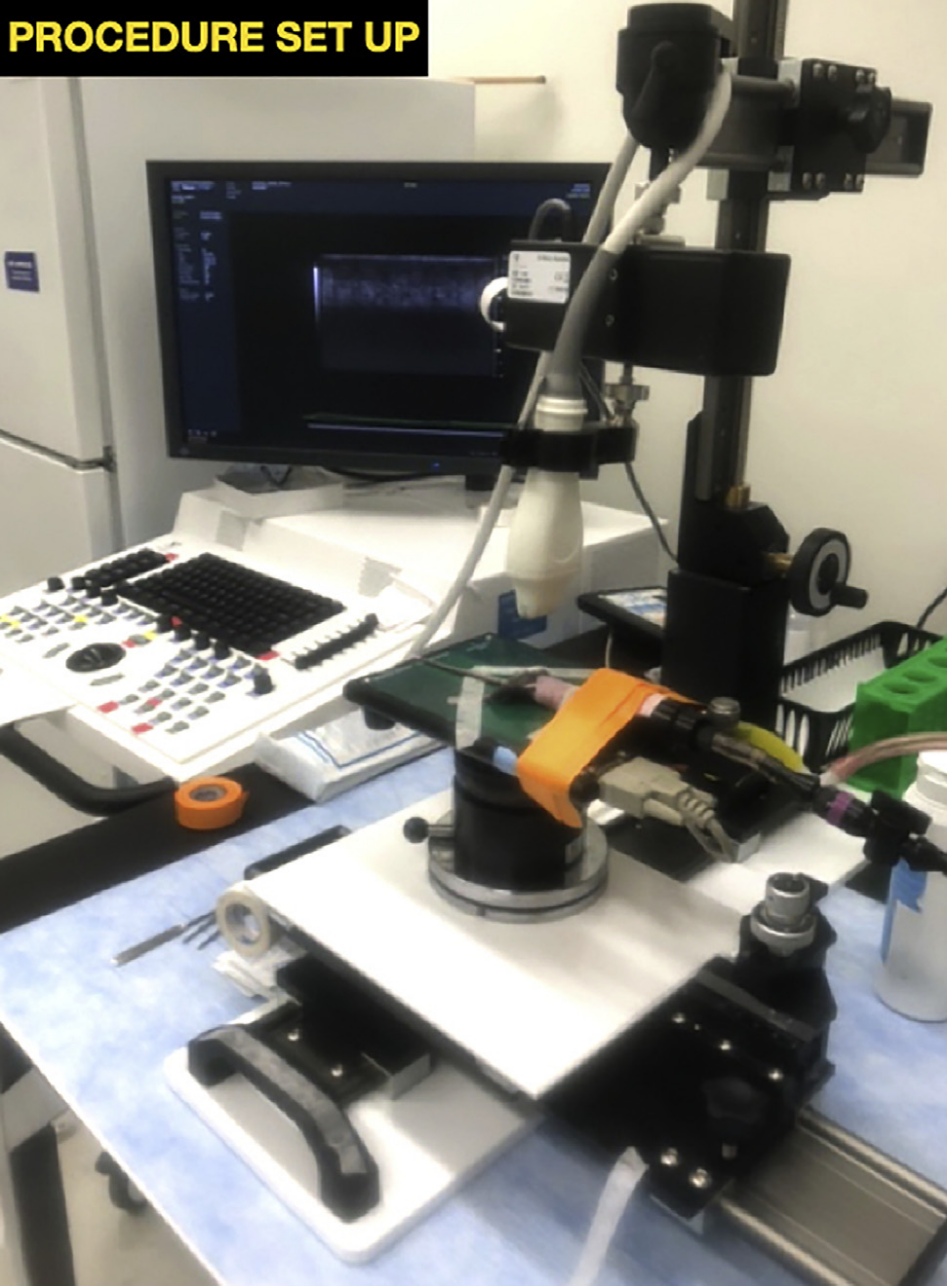
Example of properly set up ultrasound imaging and injection station with mouse secured to platform

***Note:*** All ultrasound machines have the capability of using multiple probe types (curvilinear, cardiac, linear, etc.). However, probe compatibility is dependent upon the manufacturer. A compatible MicroScan™ Transducer was used in this protocol, but another high frequency linear probe may work for this application. The procedure for switching between probes will be machine dependent.

7. Place ultrasound gel bottle on the heated platform to warm ultrasound gel prior to the procedure to lessen the risk of hypothermia for the mice.
8. Assemble required instruments.
  a. Place an absorbent pad under the Vevo® Imaging Station.
  b. Place toothed forceps, 31-gauge insulin syringes, cotton-tipped applicators, and alcohol wipes on the absorbent pad.
9. Select and mount the linear high frequency probe on the imaging station (Figure 2).

10. Orient the probe indicator toward the mouse’s left side (facing the injector).

***Note:*** This setup allows the injector to see the needle enter on the lefthand side of the screen.

11. For optimal ergonomics, position the imaging station and ultrasound screen so that the operator does not have adjust their head or body to see the screen (Figure 2).

**CRITICAL:** Ergonomics and positioning of the ultrasound screen are essential for successful ultrasound-guided procedures. The operator should not need to adjust their body position to see the screen during the procedure. Optimal positioning will be dependent on the height of the injector. Table height and positioning of the ultrasound screen relative to the injection table should be adjusted for each individual injector to minimize unnecessary movement. In other words, everything should be positioned so that once the injector has pierced the skin with the needle at the intended injection site, they only need to lift their gaze—without rotating their body or head—to see the ultrasound screen. The injector should not need to look away from the screen after that point to complete the procedure. For a sample set up, see Figure 2.

### Cell preparation for injection

**Timing: 1 h**

During this step, prepare tumor cells for injection.

12. Suspend the attached cells using preferred detachment protocol. Count the cells.

***Note:*** The B16F10-luc cells and HPAC-luc cells used in this protocol were detached from the plate using 0.25% trypsin.

13. Centrifuge cell suspension at 450 × *g* for 5 min at 4°C to minimize cell death during processing. Remove supernatant.
14. Resuspend cells in chilled PBS to wash the cells.
15. Repeat centrifugation and PBS wash (steps 2 and 3) twice for a total of 3 PBS washes.

***Note:*** Repeated washing of cells with PBS removes the FBS present in culture media prior to injection.

16. Resuspend cells in chilled PBS to the desired cell concentration. Keep cells on ice to maximize cell viability. Recommended injection volume is 30 μL per mouse.

***Note:*** It is recommended to titrate the number of tumor cells injected for each individual cell line used as the optimal time to development of metastatic lesions may differ between cell lines. The B16F10-luc cells used in this protocol were injected at a concentration of 8–10 × 10^5^ cells/mL (2.5 × 10^4^ cells in 30 μL). The HPAC-luc cells used in this protocol were injected at a concentration of 3–4 × 10^7^ cells/mL (1 × 10^6^ cells in 30 μL).

### Ultrasound-guided injection

**Timing: 10–15 min per mouse**

During this step, inject tumor cells into the portal vein at its confluence with the splenic vein (the point where the two blood vessels meet).

17. Anesthetize mice.
  a. Anesthetize one mouse at a time in the anesthesia induction chamber as per local animal review committee protocol.
    ***Note:*** For the development of this protocol, 5% isoflurane in oxygen (5 liters per minute) was used for anesthesia induction and 2.5% isoflurane in oxygen (5 liters per minute) was used for anesthesia maintenance. Prior to starting the procedure, the anesthetic chamber was topped off with isoflurane.
  b. Apply ophthalmic ointment to both eyes for anesthetic events lasting longer than 5 min.
  c. Transfer mouse to pre-heated animal imaging platform and maintain anesthesia via nose cone.
18. Secure mouse to platform using paper tape (Figures 2 and 6).

***Note:*** Each paw should be secured to the platform using a short piece of paper tape (approximately 2.5 cm). The electrocardiogram (ECG) pads can be used as a landmark, but it is not necessary to align them to the pads as the heart rate monitoring function will not be utilized.

***Optional:*** secure needle stabilization device to the ultrasound probe with the syringe-holding side facing the user (Figure 6).

**Figure 3:**
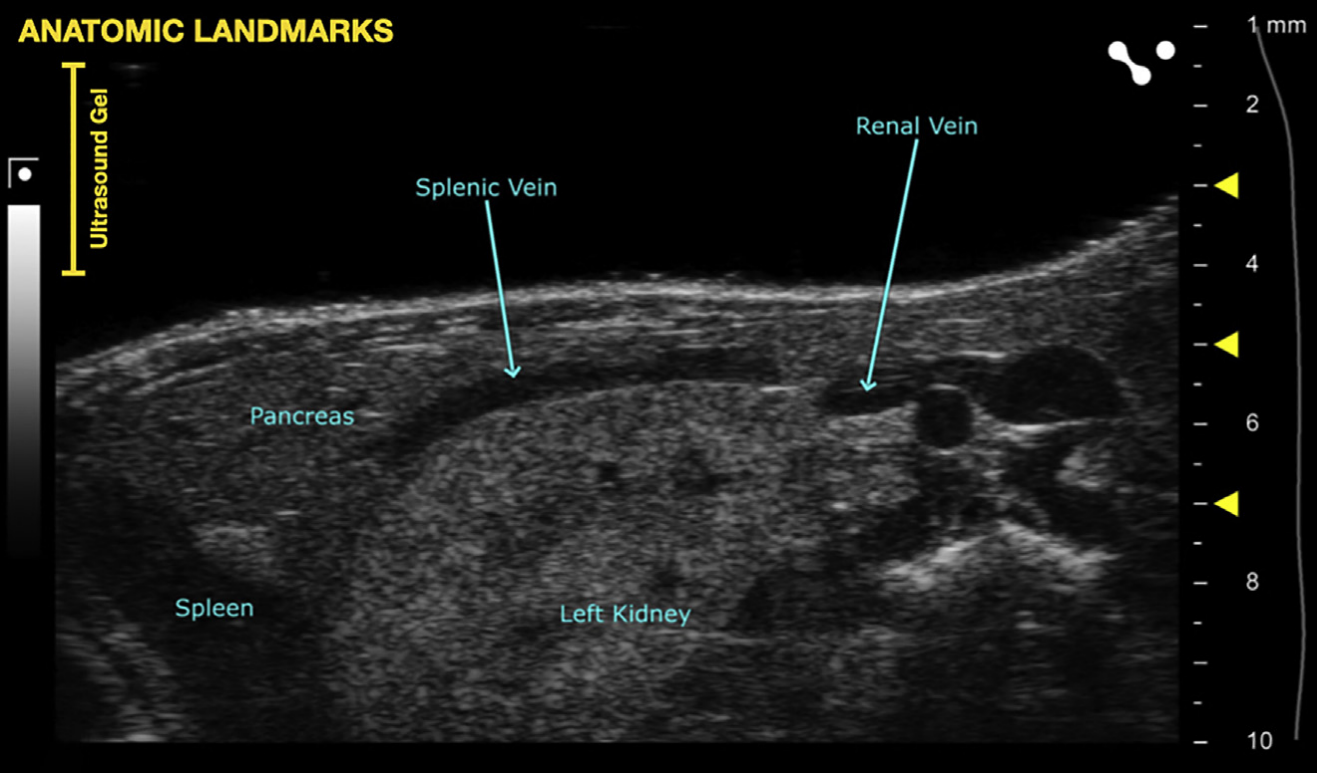
Relevant anatomic landmarks with labels
24. Scan the pancreas until you identify a blood vessel running through the pancreas. Confirm that this is the splenic vein by tracing the entirety of the vein, ensuring that it travels from the spleen all the way to the portal vein.

***Note:*** Identification of structures on ultrasound relies on the relationship between structures. For example, the left renal vein can easily be mistaken for the splenic vein if a static image is used to identify structures. Tracing the vessel will show that it emerges from the kidney and drains into the inferior vena cava. The splenic vein on the other hand, emerges from the spleen and runs through the pancreatic tissue, ultimately joining the portal vein. The portal vein can be followed cranially until it is seen entering the liver.

19. Sanitize the ultrasound probe with an alcohol wipe or 70% ethanol spray.
20. Wipe the previously shaved abdominal area of the mouse with an alcohol swab.
21. Apply a generous layer of pre-warmed ultrasound gel to the mouse.
22. Lower the probe using the height control until it meets the ultrasound gel layer. Watch the ultrasound screen for the ultrasound image to come into view.
23. Using the fine forward and backward control knobs, scan the mouse and use anatomic landmarks to identify the spleen, pancreas, and left kidney (Figure 3).

25. While the injector identifies the relevant landmarks, an assistant should prepare the cells for injection by loading 30 μL of cells into the 31-gauge insulin syringe.

**CRITICAL:** It is essential to degas the needle thoroughly as even small amounts of air can prove lethal for the mouse.

**Figure 4:**
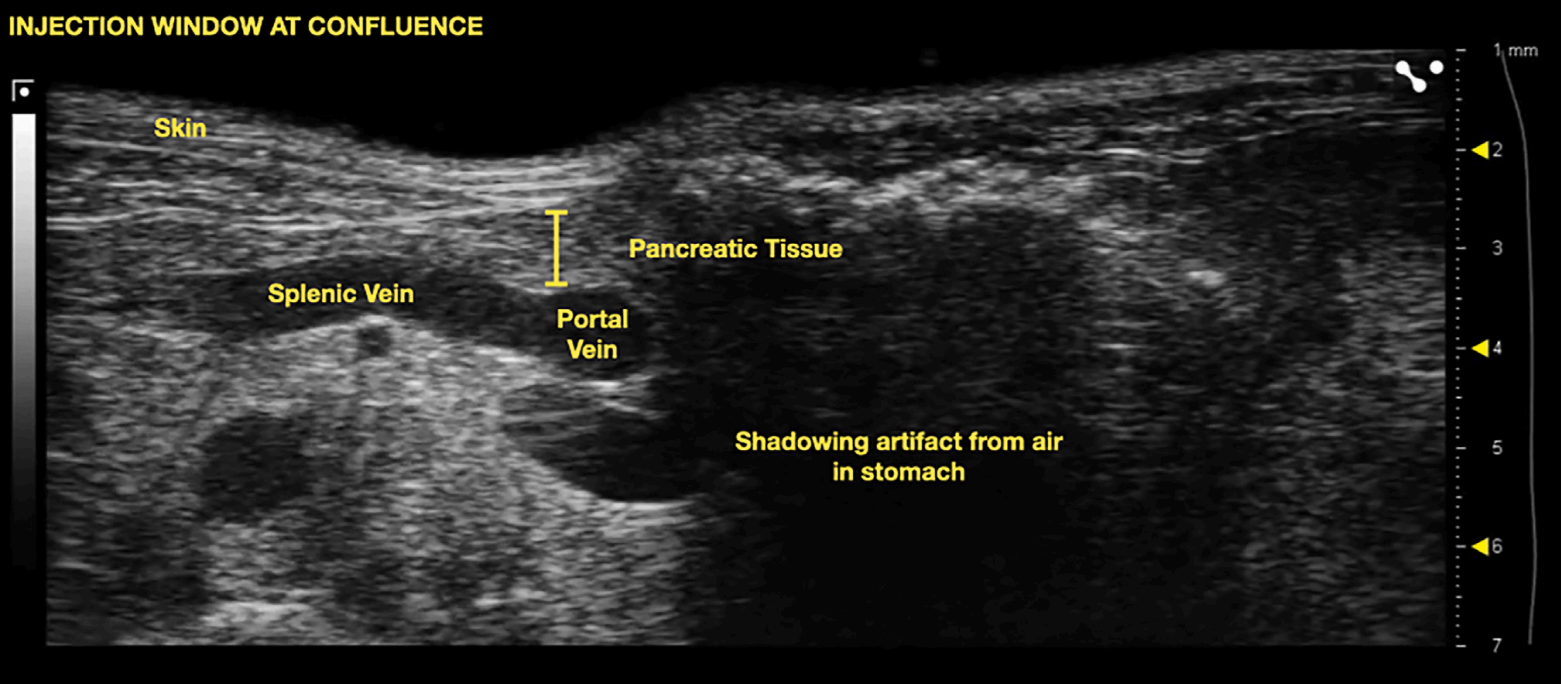
Ideal window configuration at the confluence of the splenic and portal veins

***Note:*** Adjust the gain by ensuring expected dark regions appear black on the ultrasound screen (blood and fluid should appear darker). Adjust the gain until blood within vessels is black. Similarly, adjust the depth to fill 75% of the screen with the object of interest (the confluence of the splenic and portal veins). This maximizes the quality and crispness of the image.

26. Trace the splenic vein until reaching the confluence with the portal vein. Adjust your window (Figure 4) using the forward and backward control knobs and the ultrasound depth settings.

***Optional:*** If using the stabilization device, snap the loaded syringe into place and adjust the angle of entry so that the needle tip is positioned over the target. Depending on how thick the gel layer is and how much probe pressure is applied, the probe may need to be raised using the height control to secure the needle to the device. With sufficient space, the needle can be brought into position under the probe. Then, the probe and secured needle can be lowered together into a thick cap of gel. This way, the entire length of the needle can be visualized simultaneously with the target blood vessel. Leaving insufficient space between the probe and the skin risks bending the delicate needle when attempting to position the needle.

**Figure 5:**
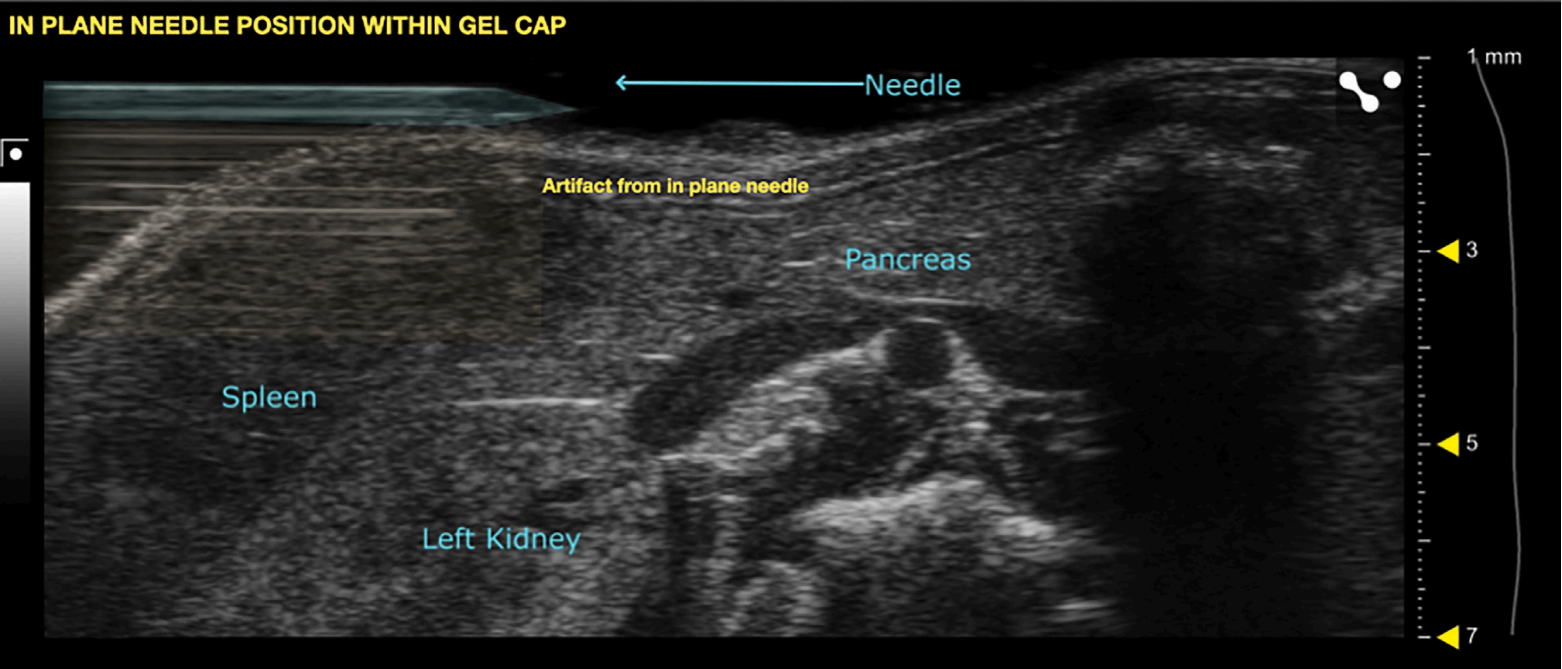
Appearance of needle relative to anatomic landmarks when positioned within gel layer using freehand, in plane technique

27. Using freehand technique, position the needle within the gel cap until it is perfectly in plane. An artifact from the needle should be visible (Figures 5 and 6).

**Figure 6:**
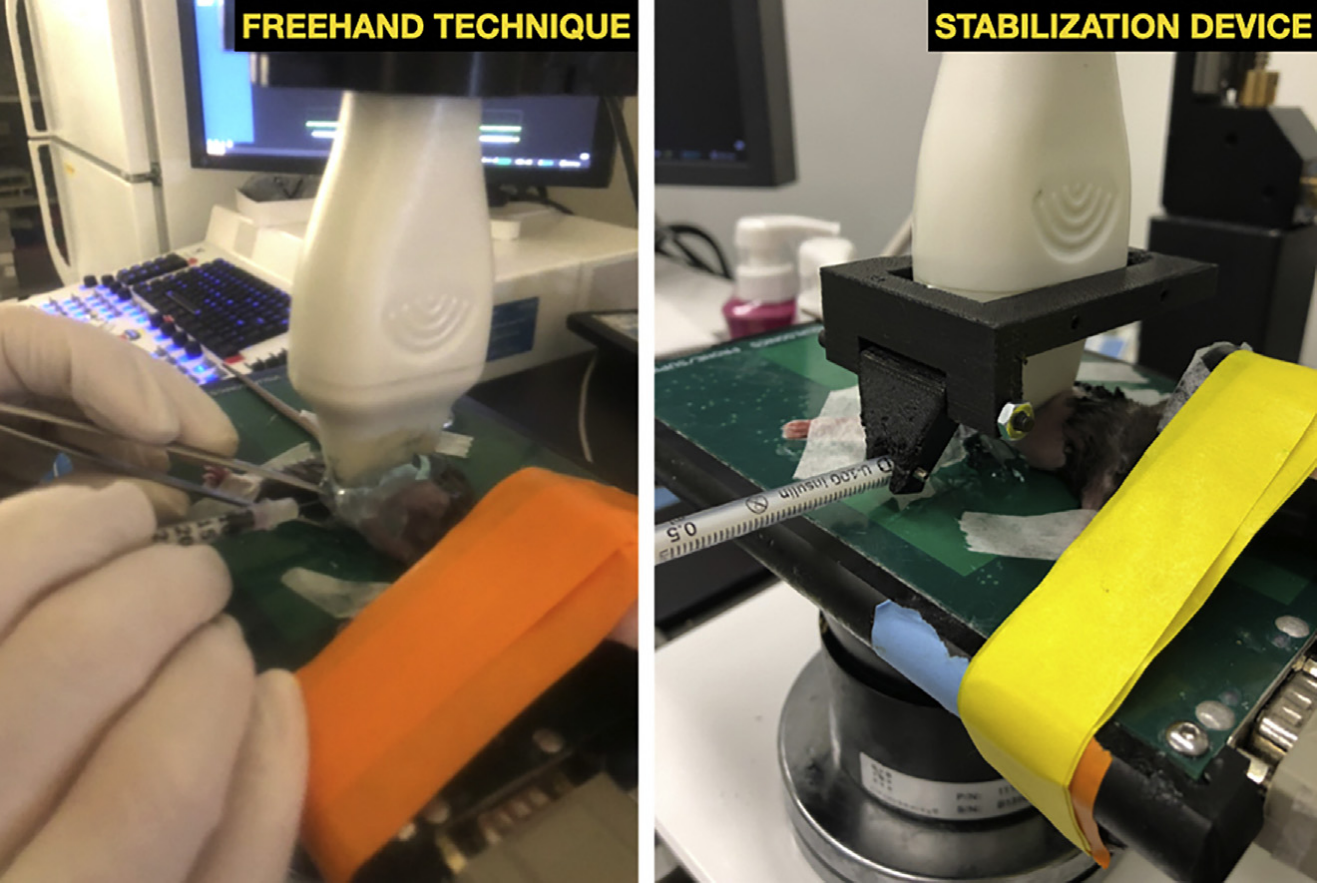
Side-by-side comparison of needle set up and positioning for the freehand and stabilization device techniques
28. Adjust the angle of entry to between 30° and 45° before advancing the needle.
29. Ensure that the needle is perfectly in plane with the ultrasound probe before proceeding.

**Figure 7:**
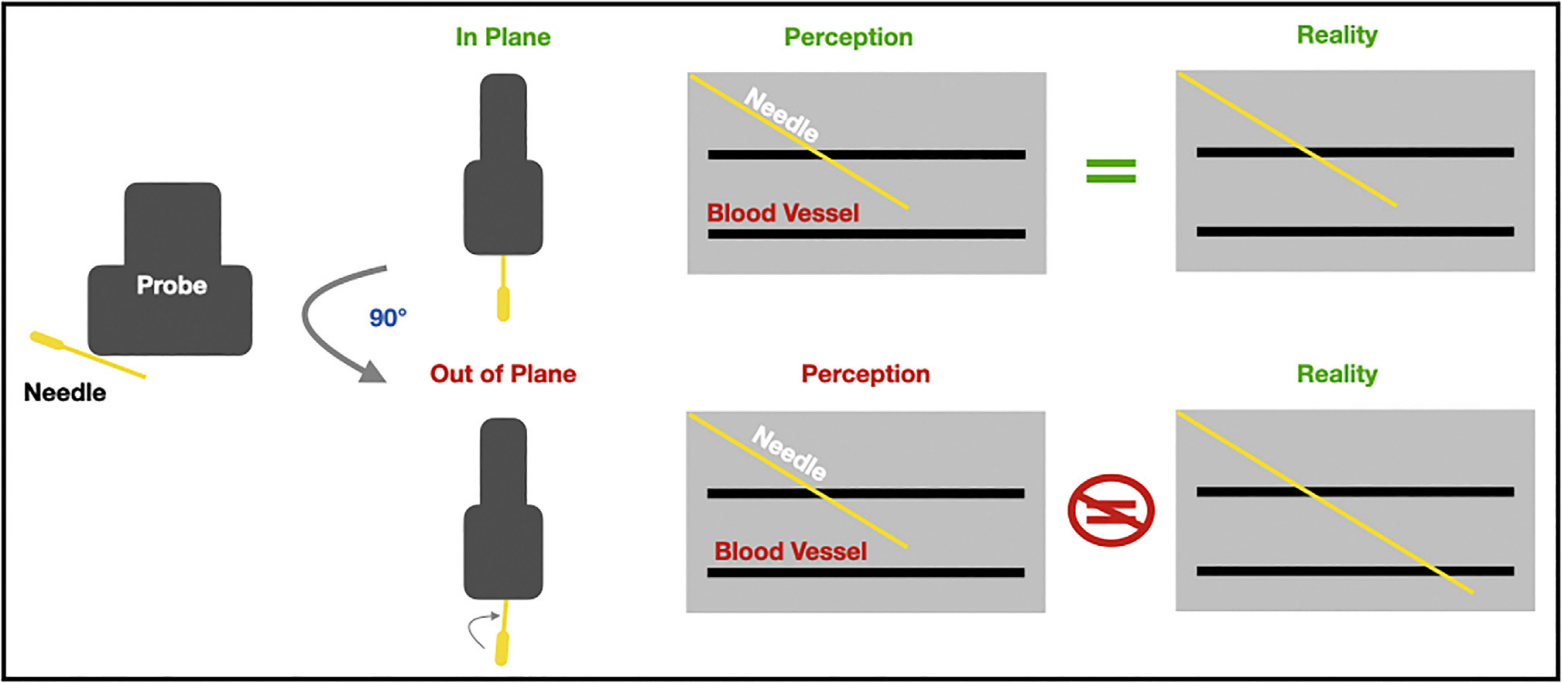
Schematic representation of in plane technique demonstrating appearance of needle relative to probe and corresponding image on the ultrasound screen

**CRITICAL:** In plane technique is the most important part of this procedure. The needle must be in plane to visualize its full length. Failure to advance the needle using in plane technique can cause the injector to advance the needle through the vessel, resulting in misinjection and/or vessel injury (Figure 7).

30. Using toothed forceps, grasp the skin lateral to the intended site of needle entry and gently pull the skin taut.

***Note:*** Creating tension allows the injector to advance the needle easily through the skin and muscle layers using their dominant hand. Once the needle is positioned at the skin and the forceps are stably grasping the skin taut, the injector should primarily look at the ultrasound screen for the remainder of the procedure.

31. Gently advance the needle using the dominant hand.

**Figure 8:**
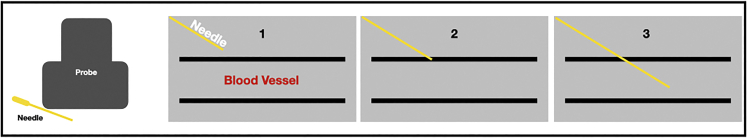
Schematic representation of appearance on ultrasound screen of needle advancing through tissue over time when using in plane technique

***Note:*** The pancreatic tissue can be difficult to penetrate. If too much pressure is applied, the needle can advance suddenly and travel farther than anticipated. If the needle is in plane with the ultrasound probe, the injector will directly observe the needle rapidly advance and simultaneously detect a loss of tissue resistance. Steady, gentle pressure permits controlled advancement of the needle through the tissue.

***Note:*** The needle stabilization device restricts lateral movement, maintaining the needle in plane with forward advancement. If using freehand technique, use fine adjustments to visualize the full needle length. The needle should not be advanced if it moves out of plane or if visualization is poor. When the needle is advanced in plane toward the desired target, the full length of the needle can always be seen and sequentially advanced (Figure 8).

32. Once through the skin and muscle layers, release the skin and drop the toothed forceps.
33. Slowly advance the needle through the pancreatic tissue (Methods video S1: Demonstration of needle advancement through subcutaneous and pancreatic tissue).

34. Confirm needle position by gently poking the vein.

**Figure 9:**
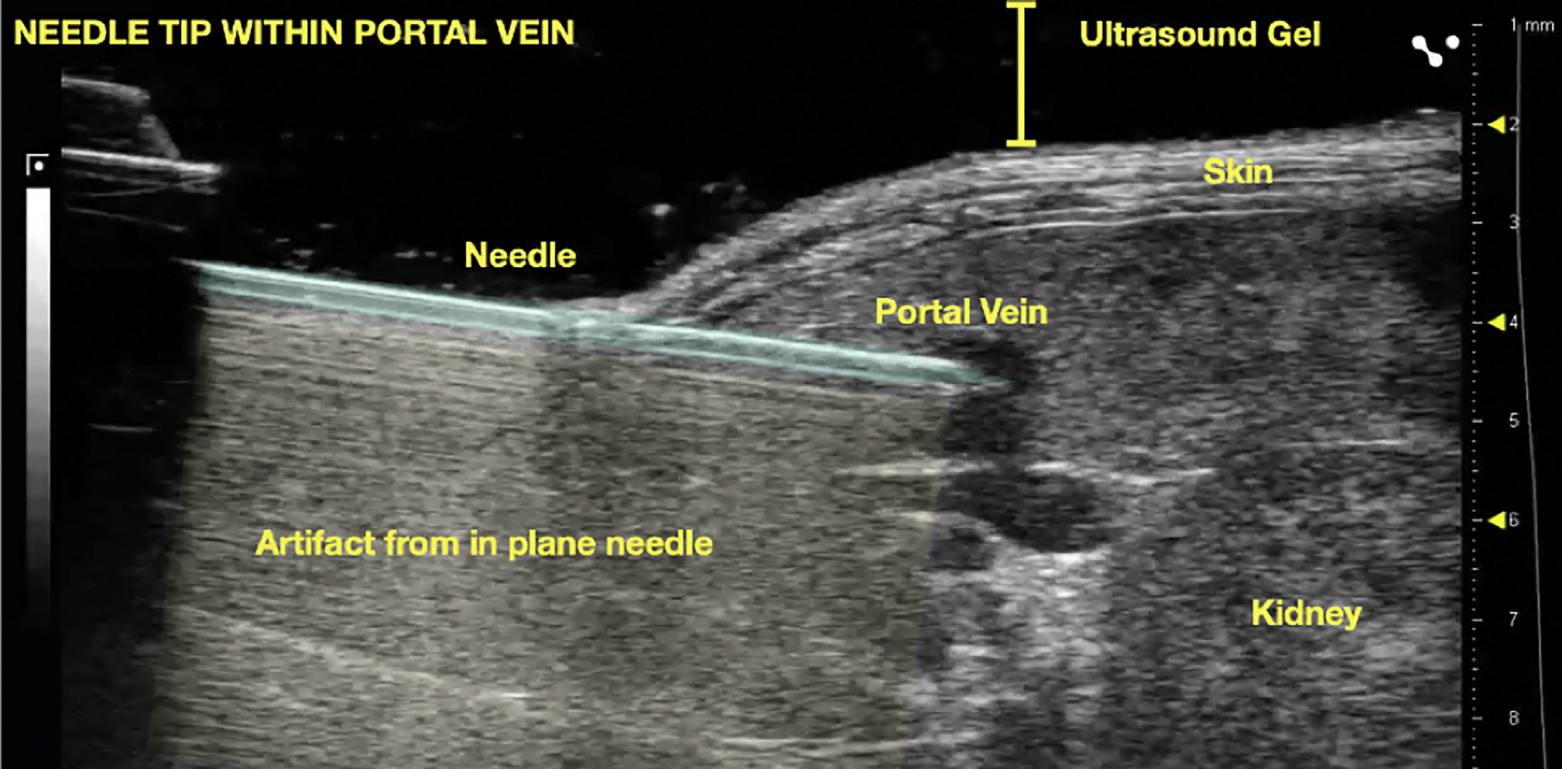
Intravascular placement of needle tip within portal vein Note artifact from in plane needle.

***Note:*** If the needle is over the vein, pressure from the needle will cause the vessel to collapse inward (Methods video S2: Demonstration of steady downward pressure applied to pancreatic tissue. Note that pressure from needle can collapse vessel). Ideally, the splenic vein is entered just distal to where it joins the portal vein so that the needle tip ends up inside the portal vein (Figure 9).

35. Apply gentle, steady pressure until the needle tip enters the vein. The tissue should visibly slide over the needle (Figure 9 and Methods video S3: Demonstration of pancreatic tissue “give” and controlled intravascular placement of needle tip).

Methods video S1. Demonstration of needle advancement through subcutaneous and pancreatic tissue, related to step 33

***Note:*** Trauma to the vessel is immediately apparent. If the vein is rapidly entered and the needle goes through the posterior wall of the vessel, extravasation of blood will occur. Even minor shearing with minimal extravasation will be visible. If this occurs, pause the procedure (troubleshooting Problem 2: Difficulty in maintaining the needle in plane).

36. Confirm intravascular placement by carefully bouncing the needle tip up and down within the vessel.

***Note:*** If the needle is within the vessel, the tip should remain inside of the vessel (Methods video S4: Demonstration of needle tip remaining within portal vein with bounce).

Methods video S2. Demonstration of steady downward pressure applied to pancreatic tissue Note that pressure from needle can collapse vessel, related to step 34

Methods video S3. Demonstration of pancreatic tissue “give” and controlled intravascular placement of needle tip, related to step 35

37. Without moving the syringe, aspirate and confirm brisk blood return in the syringe.
38. Slowly inject the cells, ensuring that no air is injected.

***Optional:*** An assistant can place the next mouse into the anesthesia induction chamber.

***Note:*** Sometimes, the needle may appear to be within the vessel when there is actually a thin layer of tissue stuck on the needle. Avoid aspirating too vigorously, particularly when the needle is not within the vessel, to prevent introduction of air into the syringe. Intravascular air injection can prove fatal to the mouse. It may be helpful for an assistant to watch the syringe as cells are injected to ensure that no air bubbles have entered the syringe.

39. After injection, apply additional probe pressure by adjusting the height control.
40. Slowly retract the needle under ultrasound guidance.
41. Release the applied pressure and observe the area to ensure the vessel has not been damaged.

***Note:*** The appearance of the vessel should be unchanged with successful injection. If misinjection into the pancreatic tissue has occurred, it will be immediately apparent at the time of injection. For example, a black fluid bubble will form outside of the vessel or there will be an abnormal accumulation of black fluid along tissue planes. Acute hemorrhage and free fluid, such as from intraperitoneal injection of the cell suspension, is not distinguishable by ultrasound; all free fluid appears black. However, intrapancreatic injection is easily distinguished from both extrapancreatic injection and vascular injury (see troubleshooting Problem 4: Extravascular injection for more detail). Blood vessel shearing and resultant extravasation of blood can be readily detected on the ultrasound screen and does not result in any observable changes at the skin injection site given the fine needle gauge and multiple layers of tissue between the skin injection site and target blood vessel.

42. Transfer the mouse to a cage on a heated platform and monitor for recovery from anesthesia. Full recovery from anesthesia should be rapid and mice should not appear to be in pain.

Methods video S4. Demonstration of needle tip remaining within portal vein with bounce, related to step 36

### Bioluminescent imaging

**Timing: 5–10 min per cage**

Duration: Every 3 days until hepatic signal increases.

During this step, perform surveillance bioluminescent imaging to detect and monitor tumor cell engraftment.

***Note:*** Time to initial bioluminescent signal varies by cell line and number of cells injected (see expected outcomes and limitations). For the purposes of defining growth kinetics during model development, mice were imaged as frequently as every 3 days. Once the growth kinetics and survival time for a given model is defined, the imaging frequency can be adjusted.

43. Anesthetize mice as per local animal review committee protocol.
44. Perform 50 μL intraperitoneal injections of D-Luciferin (50 mg/kg).
45. Acquire bioluminescent images at 5 min post-injection using the IVIS Lumina III imaging system.

***Note:*** For this protocol, we performed a four-image series with an FStop of 1.2 at 1, 5, 10, and 20 s. All images displayed in this protocol were from the first image (FStop 1 at 1 s) using a consistent color intensity scale across serial images ranging from a minimum of 1.00 × 10^6^ to a maximum of 1.00 × 10^8^.

46. Transfer mice to a cage on a heated platform and monitor for recovery from anesthesia.

## Expected outcomes

**Figure 10:**
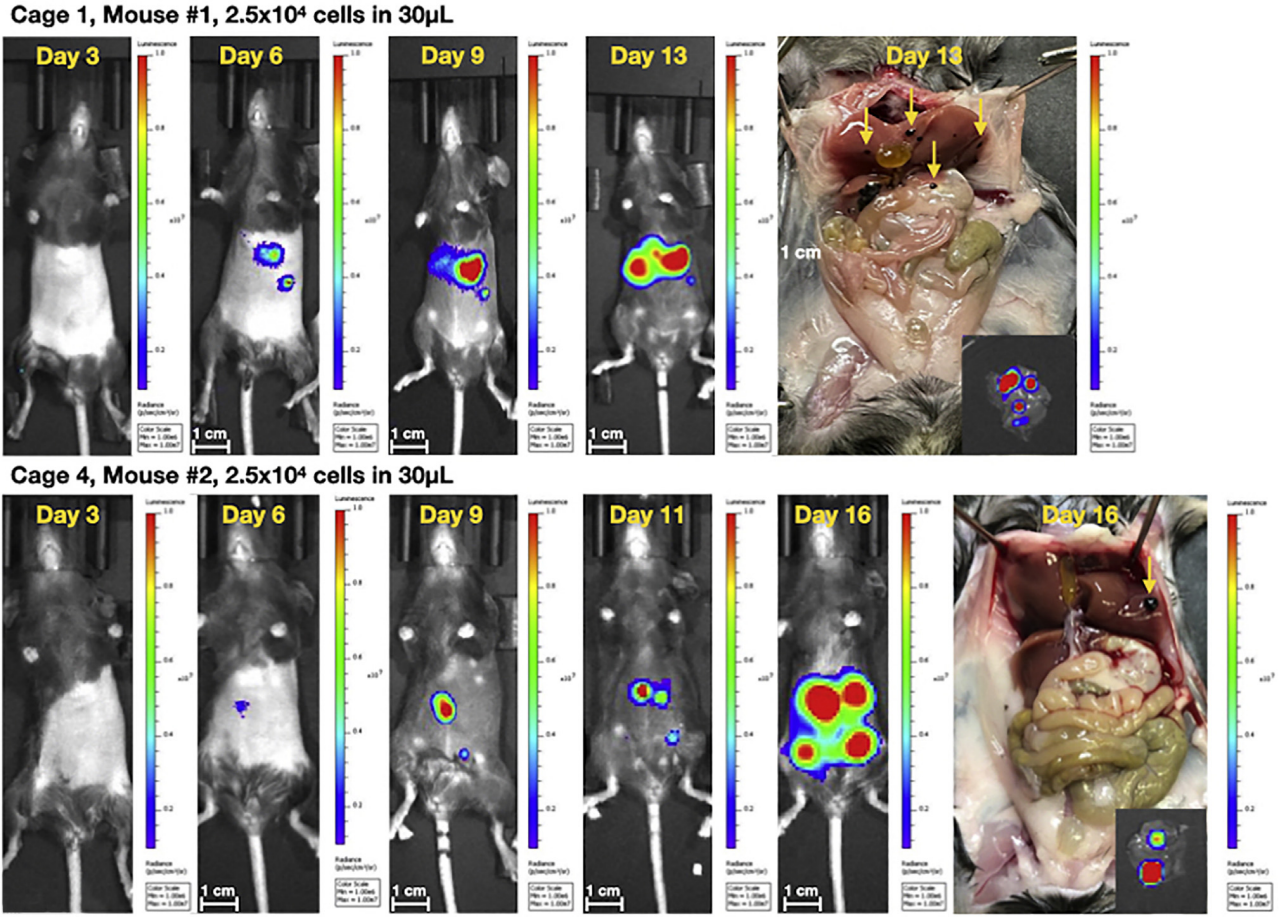
Representative sequential BLI surveillance images, necropsy photos, and ex-vivo liver BLI images highlighting variable growth kinetics in mice from the same study C57BL/6 mice were injected with 2.5 × 10^4^ B16F10-luc melanoma cells and surveilled with serial BLI (n = 15). White scale bars represent 1 cm.

**Figure 11:**
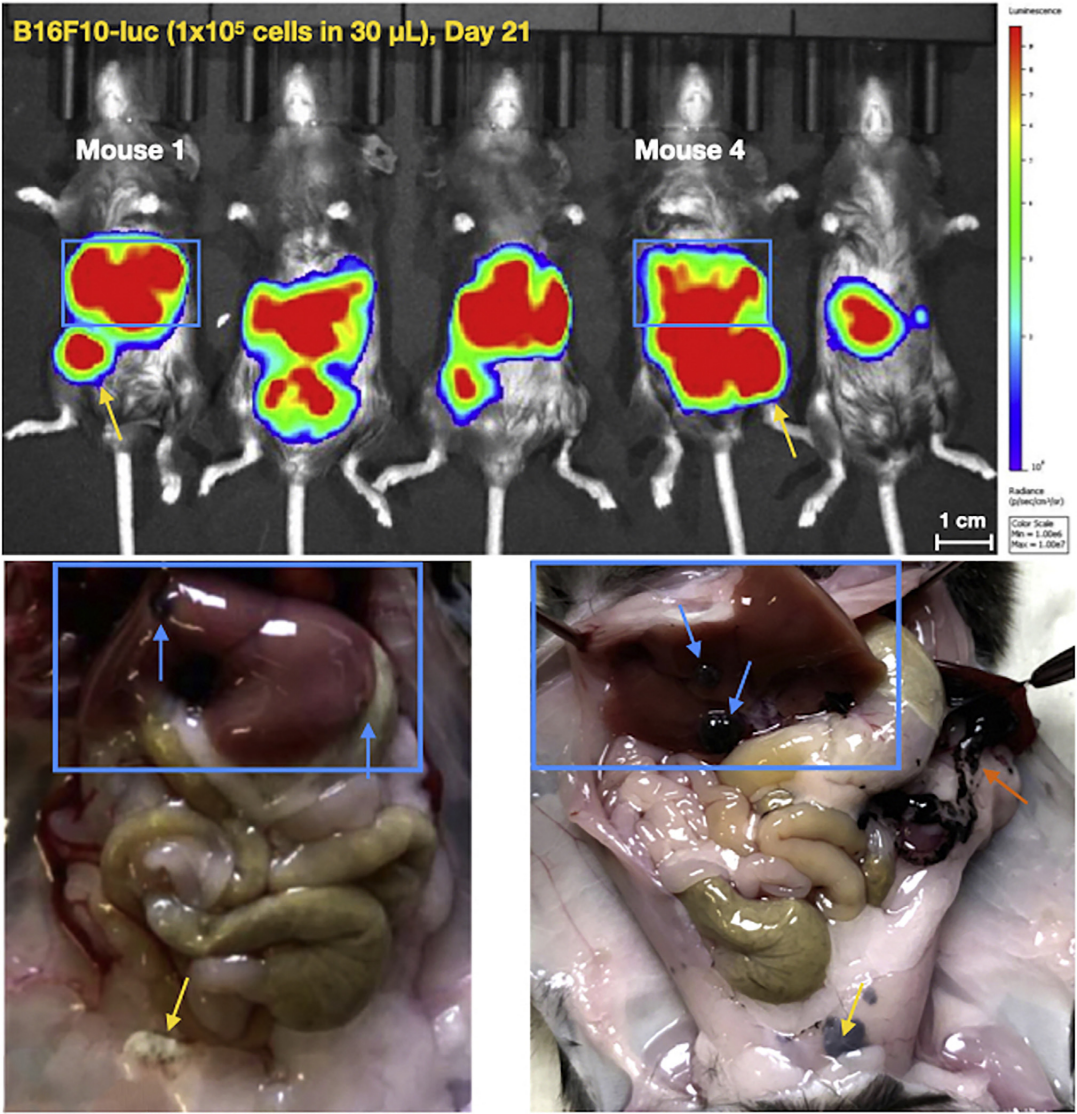
Representative sequential BLI surveillance images and necropsy photos demonstrating liver lesions in blue rectangles, fat pad lesions marked by yellow arrows and pancreatic seeding by orange arrows C57BL/6 mice were injected with 1 × 10^5^ B16F10-luc melanoma cells and surveilled with serial BLI (n = 10). White scale bars represent 1 cm.

**Figure 12:**
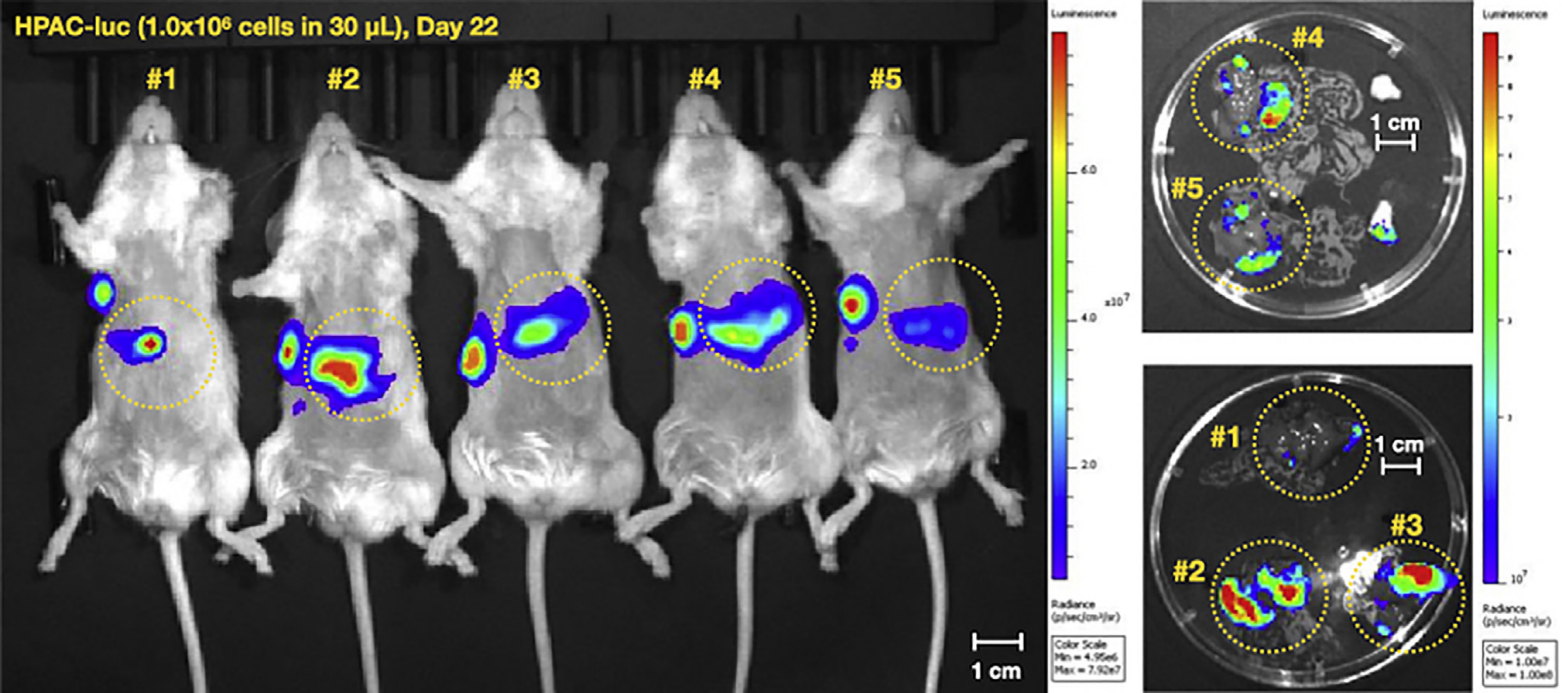
Representative sequential BLI surveillance images and *ex vivo* liver BLI images NCG mice were injected with 1 × 10^6^ HPAC-luc cells in the portal vein and 1 × 10^6^ HPAC-luc cells in the right flank subcutaneously (n = 5). All mice received both subcutaneous (positive control) and intravascular portal vein injection. Hepatic signal indicated by dashed yellow circle. White scale bars represent 1 cm.

**Figure 13:**
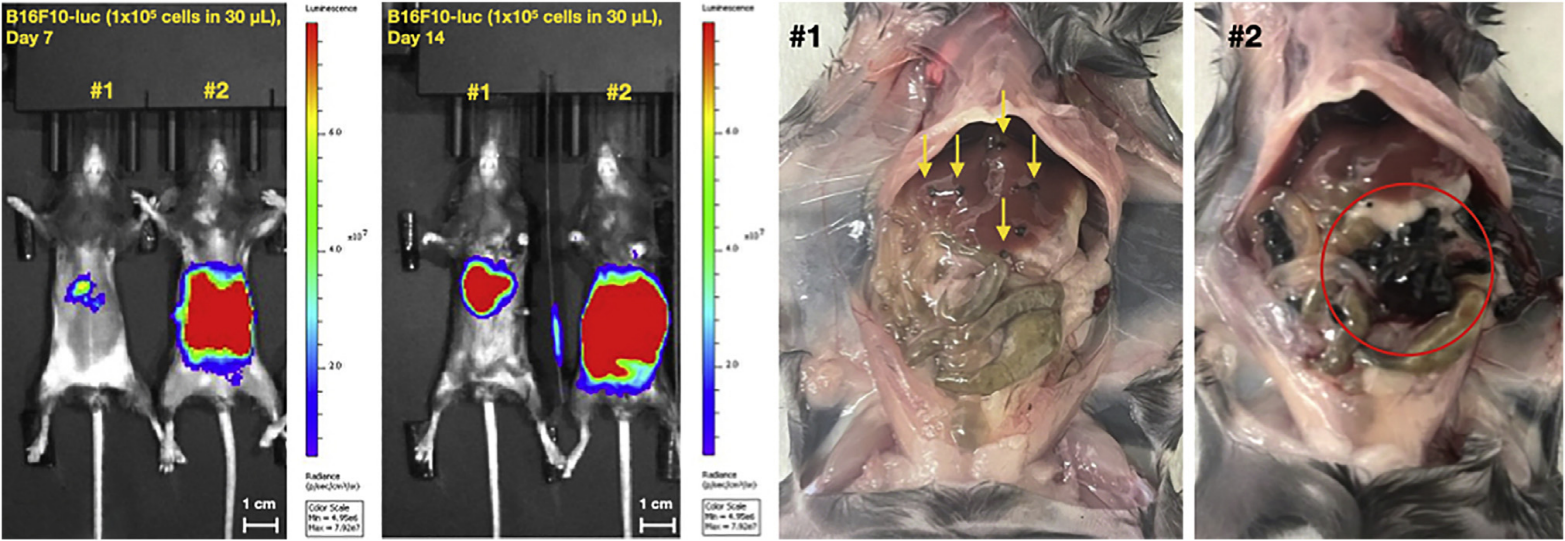
Side-by-side comparison of BLI images and necropsy photos of successful intravascular injection (Mouse number 1) vs. intraperitoneal injection (Mouse number 2) (extrapancreatic-extravascular) C57BL/6 mice were injected with 1 × 10^5^ B16F10-luc melanoma cells and surveilled with serial BLI (n = 5). White scale bars represent 1 cm.

Persistent hepatic bioluminescent imaging (BLI) signal correlates with visible, hyperpigmented lesions in the liver at necropsy in the B16F10-luc model (blue arrows in Figures 10 and 11). Liver metastases are reliably generated when the following criteria are met: 1) the syringe needle tip remains within vessel when the tip is bounced; 2) blood return with aspiration is verified prior to injection; 3) there is no vessel shearing. Time to initial hepatic BLI signal may vary even if mice are injected at the same time with the same number of cells (Figure 10). We suggest that the variability in time to initial BLI signal is cell-line dependent and can be augmented by changing the number of cells injected. When injecting 2.5 × 10^4^ B16F10-luc cells per mouse, time to initial BLI signal varied from 6 days post-injection to 19 days post-injection. 80% of well-injected mice developed persistent hepatic BLI signal and visible liver metastases at necropsy by 21 days post-injection when 2.5 × 10^4^ B16F10-luc cells were injected per mouse. This number increased to 100% when 1 × 10^5^ B16F10-luc cells were injected. When 1 × 10^6^ HPAC-luc cells were injected in NCG mice, 100% of mice demonstrated initial BLI signal by 7 days post-injection and were found to have *ex vivo* hepatic BLI signal at 22 days post-injection. Unlike mice injected with B16F10-luc cells, mice injected with HPAC-luc cells did not develop visible macrometastases. For that reason, B16F10-luc cells were chosen for further model development and validation. The propensity of B16F10-luc cells to form visible, hyperpigmented lesions was leveraged to determine the characteristics of both well-performed injections and misinjections, and to describe the expected results of misinjection (Figure 13 in limitations). Intravascular injection should not result in intraperitoneal disease (see limitations and troubleshooting Problem 4: Extravascular injection). Occasionally, trace seeding can be seen in the pancreas (orange arrow in Figure 11). In the B16F10-luc model, metastatic lesions are sometimes seen in the abdominal fat pads (yellow arrows in Figure 11), but these phenotypes do not represent extravascular injection (see limitations and troubleshooting Problem 4: Extravascular injection). Given that the number of cells injected, the cell line, and the host mouse can each influence the onset of signal post-injection and the appearance of metastases (Figures 11 and 12), optimization experiments to define growth kinetics are required for each model (see limitations).

## Limitations

As described above, time to initial BLI signal is variable. The baseline variability of the model should be determined by titrating the number of cells injected and quantifying the metastatic burden. Strategies to account for this variability include adjusting cohort sizes or pursuing an enrollment strategy after documenting an interval increase in hepatic signal. Another limitation is that growth kinetics and site of engraftment are cell-line dependent. Abdominal fat lesions were not observed with HPAC-luc lines in NCG mice (Figure 12). Tumor seeding can be optimized by adjusting the number of cells injected. Surveillance and quantification of metastatic burden is another important consideration. BLI requires the use of luciferase-expressing cell lines, but the immunogenicity of reporter genes and potential changes to the immune microenvironment are well-described.^1^^,^^2^ If using unmodified cell lines, an alternative method for quantifying metastatic burden is needed such as IHC^3^ or PCR-based methods.^4^ However, these can only be performed at experimental endpoint. When the previously outlined injection criteria are not met, extrahepatic BLI signal is detected, which correlates with intraperitoneal seeding at necropsy (red circle in Figure 13). However, even after a technically sound injection, defined as 1) fulfilled injection criteria, 2) absence of extrahepatic BLI signal and 3) absence of undesired intraperitoneal disease at necropsy, a minority of mice do not develop liver metastases. As described in the Expected Results section, engraftment rates vary with the quantity of cells injected, underscoring the importance of conducting initial cell number titration experiments. Finally, tumor cell engraftment in the pancreas is occasionally detected, but this is not unexpected given that vessels are accessed using a transpancreatic approach.

## Troubleshooting

### Problem 1

Difficulty in obtaining an injection window without overlying viscera.

After identifying anatomical landmarks, it can sometimes be difficult to obtain a safe injection window (steps 23 and 24). The most common problem is overlying small bowel that either obstructs view of structures entirely or prevents needle advancement.

### Potential solution

The easiest solution is to apply more probe pressure. Increased pressure can be accomplished by adjusting the height control, adding gel or both. Often, this solution is sufficient to displace the bowel laterally. The benefit of adding more gel is that the needle can be easily positioned within the gel cap prior to injection and is less likely to bend. Another alternative method is to use the tilt lock on the heated animal platform to tilt the mouse right side down. Then, under gentle probe pressure, flatten out the platform until reaching the starting position. This approach can “sweep” the bowel away but requires that the animal is secured to the platform with paper tape.

### Problem 2

Difficulty in maintaining the needle in plane.

Maintaining the needle in plane is technically challenging even for experienced ultrasound users and requires practice. If using freehand technique, the injector will observe that even micromovements out of plane can prevent needle visualization on the ultrasound screen (steps 27 and 29).

### Potential solution

As with any procedure, it can be helpful to rest a finger (typically the pinky) on a stable surface for additional stability. Pause the procedure when needle visualization is lost. It is not recommended to blindly advance the needle. Use fine adjustments to bring the needle back into plane (Figure 7). Once the needle is back in plane, the injector can continue advancing the needle toward the target vessel. An alternative approach to moving the needle is to maintain the position of the needle and adjust the probe slightly. It can be helpful to rotate the probe either clockwise or counterclockwise relative to the needle using the non-dominant hand. This maneuver assumes the probe is secured to the platform as in Figure 2.

### Problem 3

Needle deformation.

It is possible to unintentionally bend the needle while performing the procedure, which makes it difficult to keep the needle in plane since it is no longer straight. Typically, this happens when piercing the skin or muscle layers, or when the desired entry point for the needle is toward the middle of the probe as opposed to the lateral edge of the probe (steps 30 and 31). A 31-gauge needle was chosen for this procedure because it is finer and less likely to cause vessel trauma, but still sturdy enough to penetrate the subcutaneous tissue.

### Potential solution

Tissue forceps are the key to efficiently piercing the skin with a finer needle because they allow the injector to generate counter tension when advancing the needle through the skin. To avoid undue tension on the needle when entering the skin under the midpoint of the probe, use the forward and backward control knobs to reposition the mouse so that the target is positioned more laterally as in Figure 4. This permits a more favorable angle of entry and reduces the need to adjust the angle after piercing the skin.

### Problem 4

Extravascular Injection.

Due to pancreatic tissue texture and needle gauge, the needle can appear to be through the pancreatic tissue when it is invaginating the tissue without piercing the tissue. Furthermore, pressure from the needle can be sufficient to collapse the target vessel, making it appear that the needle has advanced farther than it has. When cells are injected without confirming intravascular placement (steps 36 and 37), extravascular misinjection can occur. Extravascular-intrapancreatic injection can be distinguished from extravascular-extrapancreatic injection by the appearance of a discrete bubble within the pancreas surrounded by a rim of pancreatic tissue. In the case of extravascular-extrapancreatic injection, turbulence from injected fluid can be directly observed along with intraperitoneal fluid accumulation.

### Potential solution

To avoid misinjection, it is important to observe the tissue slide over the needle while advancing the needle through the pancreatic tissue (Methods video S3: Demonstration of pancreatic tissue “give” and controlled intravascular placement of needle tip, related to step 35) and confirm intravascular placement prior to injection by bouncing the needle tip and aspirating blood in the syringe. Cells should only be injected after obtaining continuous blood return with aspiration. Occasionally, trace blood may be seen if the needle partially entered the vessel or has a small amount of tissue stuck on the bevel. True intravascular placement will result in steady, continuous blood return.

### Problem 5

Blood vessel shearing.

Due to the high resolution of the linear probe, even minimal blood vessel shearing can be immediately detected by ultrasound. Typically, shearing occurs when the needle advances suddenly and passes through the posterior wall of the vessel (step 35). Controlled, steady entry into the vessel while maintaining the needle in plane reduces the risk of shearing. Minor shearing is described as minimal extravasation of blood contained within the pancreatic tissue that easily tamponades with the application of probe pressure. Significant shearing is evident with more rapid intraperitoneal accumulation of blood.

### Potential solution

Minor Shearing:

• Retract the needle slightly so that the needle tip lies within the portal vein (Figure 9).
• Apply probe pressure by adjusting the height control. This can be performed by an assistant.
• Maintain probe pressure for 5 min.
• Release probe pressure and inspect area.
• If shearing is minor, blood vessel should appear intact without any evidence of ongoing bleeding or fluid collection.
• If area appears hemostatic, slowly inject cells, and observe for leakage.
• If leakage is observed, it is recommended to exclude the mouse due to the potential for undesired intraperitoneal seeding.

Significant Shearing:

• Withdraw needle completely from the blood vessel.
• Apply probe pressure by adjusting the height control. This can be performed by an assistant.
• Maintain probe pressure for 5 min.

***Note:*** Mice can tolerate a significant amount of probe pressure, but care should be taken not to apply so much pressure that venous return to the heart is obstructed.

• Release probe pressure and inspect area.
• Evaluate for hemostasis: moderate shearing may result in a small hematoma or fluid collection. When bleeding is controlled, there should not be further accumulation or expansion of the fluid collection.
• It is not recommended to attempt injection salvage or re-inject mice with moderate shearing.

## Resource availability

### Lead contact

Further information and requests for resources and reagents should be directed to and will be fulfilled by the lead contact, Timothy Donahue, MD (tdonahue@mednet.ucla.edu).

### Materials availability

This study did not generate new unique reagents.

Timothy Donahue, MD (tdonahue@mednet.ucla.edu) or Caius Radu, MD (cradu@mednet.ucla.edu).

## Data Availability

This study did not generate/analyze datasets/code. Timothy Donahue, MD (tdonahue@mednet.ucla.edu) or Caius Radu, MD (cradu@mednet.ucla.edu).
